# Pattern of *Aedes aegypti* and *Aedes albopictus* Associated with Human Exposure to Dengue Virus in Kinshasa, the Democratic Republic of the Congo

**DOI:** 10.3390/tropicalmed7110392

**Published:** 2022-11-21

**Authors:** Kennedy Makola Mbanzulu, Roger Wumba, Leonard E. G. Mboera, Jean-Marie Ntumba Kayembe, Danoff Engbu, Michael Mondjo Bojabwa, Josué Kikana Zanga, Gerald Misinzo, Sharadhuli Iddi Kimera

**Affiliations:** 1 SACIDS Africa Centre of Excellence for Infectious Diseases of Humans and Animals in Eastern and Southern Africa, Sokoine University of Agriculture, Morogoro P.O. Box 3297, Tanzania; 2 Department of Tropical Medicine, Infectious and Parasitic Diseases, University of Kinshasa, Kinshasa P.O. Box 01306, Democratic Republic of the Congo; 3 Department of Veterinary Microbiology, Parasitology and Biotechnology, Sokoine University of Agriculture, Morogoro P.O. Box 3019, Tanzania; 4 Department of Internal Medicine, University of Kinshasa, Kinshasa P.O. Box 747, Democratic Republic of the Congo; 5 Department of Veterinary Medicine and Public Health, Sokoine University of Agriculture, Morogoro P.O. Box 3021, Tanzania

**Keywords:** distribution, *Aedes aegypti*, *Aedes albopictus*, dengue, exposure, Kinshasa, Republic of the Congo

## Abstract

Dengue is a worldwide public health concern. The current study assessed the extent of human exposure to the dengue virus in relation to the distribution pattern of *Aedes aegypti* and *Ae. albopictus* in Kinshasa. Cross-sectional surveys were carried out in 2021 and 2022. The baseline entomological survey involved 19 municipalities using a grid cell sampling approach. All containers holding water were inspected for the presence of larvae in each grid. The collected larvae were kept in an insectary until the adult emergence for morphological identification. Four hundred febrile patients attending the hospital were screened for the presence of dengue antibodies (IgG, IgM) and NS1 antigen using a rapid diagnostic test (RDT) Biosynex®. Residences of positive cases were geo-referenced. We evaluated 1850 grid cells, of which 19.5% were positive for *Aedes* larvae. The positive grid cells were identified in the Ndjili (44.0%), Mont Ngafula (32.0%) and Ngaliema (26.0%), and Limete (32.0%) municipalities. The *Ae. aegypti* (11.2%) predominated in the northwestern, and *Ae. albopictus* (9.1%) appeared in the high vegetation coverage areas. Of 61 (15.3%) participants exposed to dengue, 8.3% presented acute dengue. Young, (6–17 years), male, and Mont Amba district participants were most exposed to dengue. In conclusion, dengue occurrence in Kinshasa overlaps somewhat the geographical and ecological distributions of *Ae. aegypti* and *Ae. albopictus*. Both species are not homogenously distributed, likely due to environmental factors. These findings can assist the targeted control activities.

## 1. Introduction

Dengue is among the most common febrile mosquito-borne diseases (MBD) in the world [1]. The distribution pattern of dengue infections overlaps with the geographical distribution of its competent mosquito vector species. Dengue, similar to malaria, is involved in significant morbidity and mortality rates, with an adverse socioeconomic impact in the tropics and sub-tropics [1,2]. The clinical feature of the majority of mosquito-borne viral diseases (MBVD), such as dengue, is indistinguishable from a wide range of parasitic, bacterial, and other viral febrile illnesses due to limited diagnostic tools for use in most resource-constrained settings and the similarities of their clinical symptoms [3,4]. These facts lead to misdiagnoses, and then overuse of empirical malaria and antibacterial drugs as the morbidity and mortality burden of MBVD are often underestimated, particularly in developing countries [5]. The lack or limited availability of registered vaccines and the absence of curative treatment for the majority of MBVD hamper their control. The prevention based on integrated vector control and vigorous surveillance for the early detection of MBVD can reduce the risk of outbreak occurrence [6,7]. Moreover, the public health impact of MBVD is often esteemed only during an outbreak, and surveillance is not set up during the interepidemic period. However, studies have documented the circulation of MBVD in interepidemic periods [8,9].

The outbreaks of MBVDs occur periodically across the world and tend to become progressively endemic in most of the well-known malaria endemic regions where their principal vector, *Aedes aegypti*, and *Aedes albopictus* coexist [10,11]. The global epidemiological standing over time identified the Democratic Republic of the Congo (DRC) as a country in which MBVDs, such as dengue, are an emerging or re-emerging disease. Dengue virus has been present in many provinces of the DRC for many decades with increased activity in recent years [12]. The recent report of dengue fever occurred during the chikungunya outbreak in 2012 in Kinshasa and later in 2016 [13,14]. The four serotypes of dengue have been documented in the DRC [15,16].

The *Aedes aegypti* is the main urban vector for arboviruses such as dengue, yellow fever, chikungunya, and Zika. Except for yellow fever, the Asian native mosquito *Ae. albopictus*, which has invaded countries all over the world, is another competent vector for the transmission of these MBVDs [17]. The bio-ecology of these vectors is closely linked to climatic conditions (tropical and subtropical); environmental factors, such as the multiple natural or artificial water courses or containers; lack of proper waste management; and anthropogenic activities [18,19]. These conditions and factors are present in sub-Saharan Africa and lead to multiple MBVD outbreak occurrences. In urban areas, both species are mostly bred in uncovered domestic water storage units or any discarded container holding water. Moreover, in rural or forest areas, they are commonly bred in tree holes. The female adult mosquito is a blood-feeding insect that bites human during the daytime and, once infected, can successfully transmit and spread viruses [20,21]. In recent years, *Ae. albopictus* has been established in many places in Kinshasa [22,23,24] and is spreading to other provinces. It was recently documented in Tshuapa, a region located about 770 km from Kinshasa [25]. Therefore, both competent vectors of dengue, *Ae. aegypti*, and *Ae. albopictus* are present in the DRC. Little is known about their spatial distribution pattern and role in the spread of different MBVDs such as dengue in the DRC, which has recently experienced multiple chikungunya and yellow fever outbreaks [13,26,27,28].

Dengue is an MBVD that has rapidly spread to all regions of the world in recent years. Today, almost half of the global population is at risk of dengue [1]. The clinical signs and symptoms include fever, joint pain, muscle pain, retro-orbital pain, and rash. Sometimes, the patients can present severe hemorrhagic disease and dengue shock syndrome, as well as respiratory and gastrointestinal manifestations [29]. The diagnosis of dengue infection can be performed serologically by enzyme-linked immunosorbent assays (ELISA), which may confirm a recent or past infection based on the presence of IgM or IgG antibodies. The viral ribonucleic acid detection by reverse transcriptase–polymerase chain reaction (RT–PCR) remains the gold standard. However, these expensive and sophisticated methods require appropriate equipment and experienced staff. The choice of the method to use should consider the time of patient presentation. RDT (rapid diagnostic test) dengue remains the convenient first choice in dengue-endemic regions and locations without adequate laboratory facilities [1,30]. The recent global spread of dengue has implied an urgent need for accurate, not time-consuming, and low-cost disease diagnostic methods such as RDT that could be easy to use even in resource-limited settlements [31], such as the DRC.

There is an important need to conduct a local investigation to gain insight into the extent of dengue in a country such as the DRC. The better identification of risk areas can rationally assist healthcare authorities to prioritize more efforts of the control activities at higher risk exposure areas. The primary objective of the current study was to assess the entomological risk related to human exposure to dengue regarding the abundance and distribution pattern of *Ae. aegypti* and *Ae. albopictus* in Kinshasa, the Democratic Republic of the Congo.

## 2. Methods

### 2.1. Study Area

**Figure 1:**
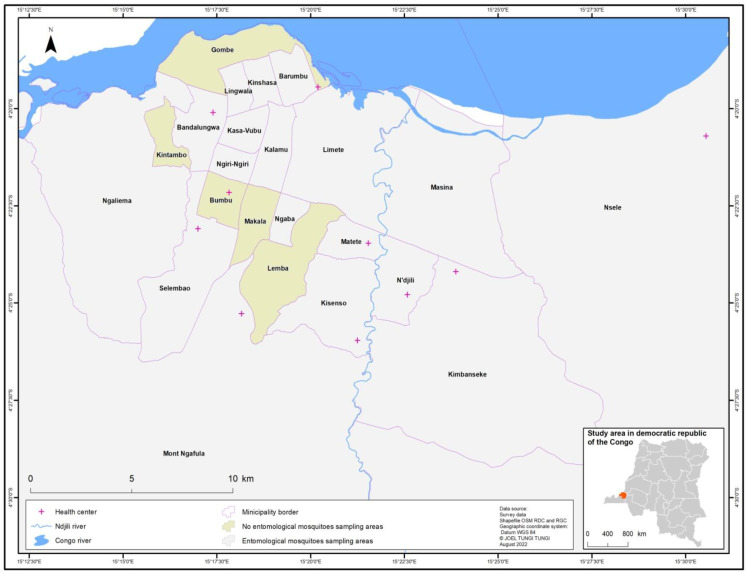
Map of the study areas.

The Democratic Republic of the Congo (DRC) is geographically the largest country in sub-Saharan Africa (SSA). This study was carried out in Kinshasa located on the south riverside of Congo River, directly beside Brazzaville, the capital of the Republic of the Congo. Kinshasa as the capital is one of the 26 provinces of the DRC. It covers a surface area of 9965 km^2^, over 90% of which is rural. The climate is categorized into two seasons, a dry season which runs from mid-May to mid-September, and a rainy season from the second half of September to the first half of May. Kinshasa has an estimated population of 17 million inhabitants. Administratively, Kinshasa is divided into 24 municipalities which are grouped into four districts, namely Tshangu, Mont Amba, Funa, and Lukunga. (i) The Tshangu district is located in the eastern part, (ii) Mont Amba is located in the west of Tshangu and lays on the south to north of Kinshasa, (iii) Funa is located in the center to the southwestern part, and (iv) Lukunga is situated in the western part. The urbanization of Kinshasa is not homogenous, even within the municipality. In general, Kimbanseke, Nsele, Maluku, Kisenso, Selembao, and big parts of Mont Ngafula and Ngaliema are considered suburban areas [32]. Mosquitoes were collected in 19 municipalities from the four districts and a hospital-based serosurvey covered the entirety of Kinshasa city, as shown the Figure 1.

### 2.2. Study Design and Period

*Entomological investigation:* The cross-sectional design was used to investigate the geographical and seasonal distribution patterns and the abundance of *Ae. aegypti* and *Ae. albopictus* in Kinshasa. The study was carried out from February 2021 to August 2022. The sampling was not performed simultaneously across all grids. In 2021 and 2022, 11 and 8 municipalities were surveyed, respectively. The grid technique was used for the mosquito sampling plan. Each municipality was divided by a grid whose cell measured 100 m × 100 m on a side. Then, 100 grid cells were randomly selected per municipality. All selected grids within a municipality were concomitantly inspected once during each season. To locate the cell during the survey, a geographic coordinate was generated at the center of each of the selected cells. In wide municipalities such as Mont Ngafula, Ngaliema, Nsele, and Kimbanseke, some quarters were excluded from the sampling plan, and in Maluku, only the Kimpoko neighborhood was involved in the study with 50 selected grid cells. All natural containers (bamboo, plant leaves), artificial containers, and containers used for domestic purposes (bottles, bowls, buckets, jars, cans, tires, and any other item) holding water were inspected for the presence of larvae or pupae [33]. In addition, the information about the water supply system availability was recorded. The larvae and pupae were reared into adults [24]. The adult *Aedes* mosquitoes were identified to species using a pictorial key [34].

*Investigation of human exposure to dengue:* A cross-sectional survey was carried out from June to July 2022 in the four districts of Kinshasa. The febrile patients aged above 5 years attending healthcare facilities were included. The healthcare facilities were selected to cover the whole of Kinshasa with the expectation of including 100 participants in each district, totaling 400 patients.

In the eastern part, Centre Médical Evangélique Révérend Mbakani (Ndjili), Hope Clinic (Kimbanseke), and Centre médical Promedis (Nsele) for residents of Tshangu were included. In the northern part, Centre Médical Militaire de référence de forces navales (Gombe) was included. In the southeast, Centre de santé de reference maziba (Matete), Centre de santé de reference sainte Ambroise (Kisenso), In center Hopital general militaire de référence Kokolo (bandalungwa), Centre médicalmère et enfant de Bumbu (Bumbu), and Hopital du monastere (Mont Ngafula) were included. In the western part, the healthcare facilities involved in the current study were included.

In the current study, immunochromatographic RDTs, which detect the presence of non-structural protein 1 (NS1) and play an important role in the early diagnosis of dengue fever (up to 7 days from the onset of symptoms), were used together with another type of RDT which detects antibodies of IgG and IgM to assess the extent of the human exposure to dengue. From the data in the literature, the Biosynex® RDT showed the best performance among the five evaluated RDTs [35], and such performance was confirmed afterward by the study carried out in New Caledonia that reported the sensitivity and specificity of the NS1 antigen RDT Biosynex® of 79.9% and 96.2%, respectively [36].

All participants were screened for dengue antibodies (IgG and IgM) using 10 µL of the blood sample and 3 drops of the buffer of RDT Biosynex dengue Ab BSS® kit from lot KDE220331 (Biosynex Swiss SA, Rue de Romont 29–31-CH-1700 Ftibourg-Switzerland) according to the manufacturer’s instructions, with a reading time of 15 min. The RDT Biosynex dengue Ag BSS® lot DEG2201001-S that detects the dengue NS1 antigen, with high specificity for acute dengue, was used for screening all dengue IgG- and IgM-positive cases, and some negative samples following the manufacturer’s guidelines. Malaria was also screened among the participants using a RDT for *Plasmodium falciparum* ParaHIT®. In addition to the sociodemographic characteristics, the clinical symptoms were recorded for each participant on a questionnaire. All residences of the positive dengue cases were visited afterward. Then, geographical coordinates were taken using a GPS gamin device and the entomological investigation was undertaken.

**Figure 2:**
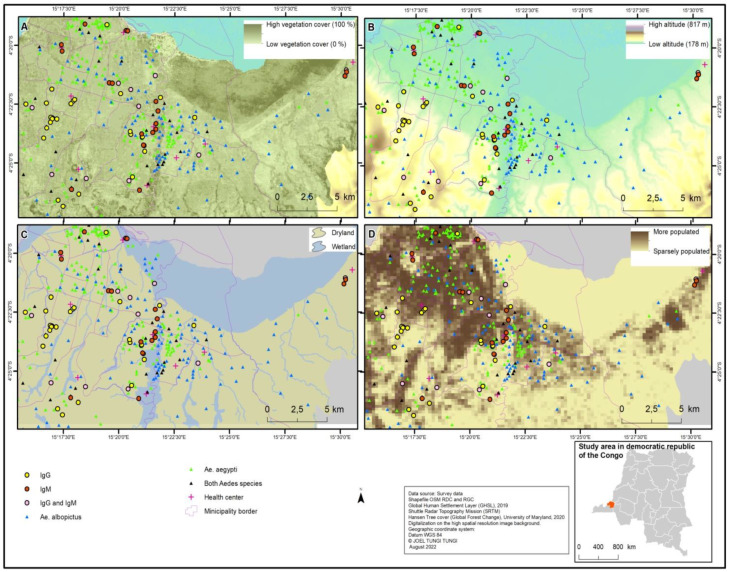
Distribution pattern of *Aedes aegypti*, *Aedes albopictus*, and human exposure to dengue virus in relation to environmental parameters of Kinshasa. (**A**) Distribution pattern of *Aedes aegypti*, *Aedes albopictus*, and human exposure to dengue virus in relation to Kinshasa’s vegetation coverage. (**B**) Distribution pattern of *Aedes aegypti*, *Aedes albopictus*, and human exposure to dengue virus in relation to different states of altitude in Kinshasa. (**C**) Distribution pattern of *Aedes aegypti*, *Aedes albopictus*, and human exposure to dengue virus in relation to the humid and non-humid areas of Kinshasa. (**D**) Distribution pattern of *Aedes aegypti*, *Aedes albopictus*, and human exposure to dengue virus in relation to the demographical density of the population in Kinshasa.

*Ecological data:* With the collaboration of the Central African Satellite Forest Observatory (OSFAC), Kinshasa, the geospatial maps were performed using ArGis 10.8 (https://www.esri.com/, accessed on 12 September 2022). Three environmental parameters, including the vegetation cover, humidity (proximity to water bodies), and altitude of the municipalities, were used to discriminate the different ecological classes that can have a negative or positive impact on the presence of mosquitoes and dengue exposure. The three parameters varied as follows: (i) Vegetation cover varied between zero soil (0%) and area covered by vegetation (100%), (ii) the elevation varied from 178 to 817 m, (iii) the data on Kinshasa water bodies helped to identify humid areas, and (iv) the demography varied by population density. The data came from field collection, and the geographical coordinates of positive grid cells to *Ae. aegypti* and *Ae. albopictus* together with those of the positive cases of dengue exposure were associated with different ecological parameters to produce maps (Figure 2).

### 2.3. Statistical Analysis

Data analysis was performed using Epi Info version 7. The frequencies and percentages were calculated for categorical variables. Odds ratios (ORs) and 95% confidence intervals (CIs) were calculated and *p* < 0.05 was considered statistically significant. Multivariate logistic regression models were constructed to identify predictor factors.

### 2.4. Ethical Consideration

The study protocol was approved by the Ethics Committee of the School of Public Health, University of Kinshasa, DRC (reference number: ESP/CE 058/20199). Participants or parents/guardians of participants under 18 years old provided written informed consent before undertaking study-specific procedures. They were informed about the objectives, risks, and benefits of participating.

## 3. Results

Of 1850 grid cells investigated during the whole study period, 361 (19.5%) were found positive for larvae, mostly throughout the rainy season (18.9%). The percentage of positive grid cells varied according to the municipality and was high in Ndjili (44.0%), Mont Ngafula (32.0%) and Ngaliema (28.0%), and Limete (32.0%) (Table 1). The positive containers ranged from discarded (23.6%), domestic (34.5%), and tires (38.0%) to natural bamboo (3.9%). However, the frequency of the positive container types varied according to location. The containers for domestic use predominated in Mont Ngafula (76.1%), Kisenso (73.0%), and Maluku (62.5%); the discarded containers were common in Barumbu (69.2%) and Kinshasa (65.2%); the tires were distributed everywhere, but predominated in Kasa-Vubu (78.3%) and Ngaba (69.2); and the bamboo was mainly recorded in erosion and humid areas of Nsele (25.0%), Kimbanseke (17.6%), and Kalamu (10%) (Table 2). In the sampled grid cells in the rainy and dry season, *Ae. aegypti* was recorded in 11.2% and 0.7% grid cells, respectively, but it predominated in the north and west sites. *Aedes albopictus* was recorded in 9.1% and 0.8% in the rainy and dry season but predominated in the south and east of Kinshasa (Table 3) where the vegetation coverage appeared relatively high (Figure 2).

### Eneral Characteristics of the Study Population

Four hundred participants, aged from 6 to 80 years, with an age mean of 32.8 years (SD = 17.7 years), were included in this study. The participants aged above 17 years were 78.8% male, and females represented 61.2%. Overall, 34.7% of participants had no occupation, 58.7% were unmarried, and 41.0% attended secondary school (Table 4). Febrile study participants claimed that they had at least one of the following symptoms: headache (51.4%), joints pain (28.1%), muscle pain (18.8%), vomiting (11.3%), or diarrhea (5.5%). The clinical characteristics of the participants are shown in Table 5.

Table 1 provides the number of grid cells positives for at least one container with immature stages of *Aedes* spp. per 100 inspected grid cells in the municipality. The numbers of the positive grid cells are provided for each season, the overall study period, and area. A positive grid cell refers to a grid with at least one container with immature stages of *Aedes* spp.

Table 2 provides the number of positive container types from each municipality according to the rainy and dry seasons. N is the total number of containers positive for immature stages of *Aedes* spp. per 100 inspected grid cells. A positive container refers to a container found with at least one larvae or pupae of *Aedes* spp.

Table 3 reported the frequency of the occurrence of *Ae. aegypti* and *Ae. albopictus* per 100 grid cells inspected in each municipality and for the whole study area according to the season.

Overall, 61 (15.3%) participants were exposed to dengue. The frequency of dengue IgG and IgM antibodies was, respectively, 9.8% and 7.8%; of these, 2.5% concomitantly expressed both IgM and IgG. Two additional cases of dengue were detected using the NS1 dengue RDT among the seronegative cases; therefore, the frequency of acute dengue (IgM- or NS1-positive) was evaluated at 8.3%. The frequency of seropositivity was, respectively, 6.0%, 14%, 17.0%, and 22% in the Tshangu, Lukunga, Funa, and Mont Amba districts. Limete (29.7%), Mont Ngafula (26.1%), Selembao (24.1%), and Kisenso(21.4%) were the municipalities with a high record of seropositive cases (Table 6 and Table 7).

In the multivariate analysis, the male participants (aOR: 2.4, *p* = 0.0271) were more exposed to dengue than females, and those aged 18–34 years (aOR: 0.8, *p* = 0.0252) and 52–80 years (aOR: 0.3, *p* = 0.0489) were less exposed to dengue compared to participants aged 6–17 years. The level of exposure was three-times higher in the Mont Amba district (aOR = 0.0123, *p* = 0.0123) than in the Tshangu district (Table 8). Figure 2 shows the geographical distribution of dengue exposure and their vector concerning the study’s environmental parameters.

## 4. Discussion

The overall results from the present study reveal that *Aedes* mosquitoes are most abundant during the rainy season. Most larvae habitats are anthropogenic due to the management of water stocking, and the mismanagement of environmental and ecological factors are among the drivers of mosquito occurrence. In suburban municipalities which lack permanent water supply sufficient for domestic uses, most larvae habitats are the domestic containers used to stock water. In most resident plots from these municipalities, people set a outdoor big water container or multiple small containers to collect the rainwater; this observation might explain, in part, the abundance of mosquitoes during the rainy season in these areas. Even in the dry season, the outdoor containers are used to stock water, but some of them remain uncovered. This observation is more documented in the areas with small house sizes, likely due to limited space indoors, and in places where construction activities are undertaken, as most people prefer to build during the dry season. Inadequate and irregular water supplies are known as driving factors of mosquito abundance in many locations [37,38]. Although discarded containers are found everywhere in Kinshasa, they were predominant in most populated areas, as well as the advanced urbanization or uncontrolled urbanization areas such as Matete, Kasa-Vubu, Barumbu, Limete, and Kinshasa. The tires were a larvae habitat widely distributed over the whole of Kinshasa, but mostly in the municipalities where automobiles garage activities or related activities were practiced such as in Ndjili, Kasa-Vubu, Ngiri-Ngiri, and Ngaliema (Delvaux), and in municipalities with relatively high socioeconomic status such as Lingwala, Limete, Kasa-Vubu, Masina (sans fil), Barumbu, and Bandalungwa, likely because people from rich municipalities can afford a vehicle or car and can change damaged tires and keep them outside their homes. In addition, the tire trade is common in those areas. The findings from the studies in Pakistan and Iran underline the roles of the tire trade in spreading dengue vectors in new areas previously not known endemic to dengue [39,40]. The bamboos were larvae habitats mainly in municipalities that lay on or crossed by water bodies and in highland areas.

These municipalities face soil erosion, and bamboo is planted to stabilize the soil and control the erosion. However, bamboo is known to be one of the most productive larvae habitats due to its water salinity and dissolved oxygen [24]. In addition, some people have created large ground pits to retain rainwater to prevent landslides in their neighborhoods. Such practice is contributing to increasing mosquito production in these areas. Both *Aedes* species are widely distributed in Kinshasa, but not homogeneously. *Aedes albopictus* is slightly predominant in suburban areas, likely due to the high level of vegetation coverage. This observation is inconsistent with the findings from the literature [41,42].

Dengue contributes to the burden of febrile illness in Kinshasa. However, due to lack of attention that it has received by medical practitioners and the lack of appropriate diagnostic tools in most areas of sub-Saharan African countries, it remains underdiagnosed [43]. Findings from different studies have reported that NS1 RDTs have high specificity; their sensitivity can be affected by the time of sampling and dengue serotypes [44,45]. However, this performance of RDT in the acute phase of dengue is enhanced once it is combined NS1 with IgM/IgG RDT [46]. Indeed, the use of these combined types of RDTs provides an advantage to the study participants regarding information about chronic and active infections. IgM is produced early during the acute phase of the disease and disappears very soon. However, IgG is produced later and can remain longer; therefore, it can reveal a past exposure [1]. The current serosurvey was carried out during the dry season, and the presence of the dengue IgG was recorded in 38 participants. High-level exposition to dengue may occur more frequently during the rainy season, as *Ae. aegypti* and *Ae. albopictus* are abundant during that period. Detection of the IgM antibody and NS1 among participants highlights the importance to consider dengue as the major etiology of febrile illness in Kinshasa, where only 29.0% of participants had positive results of malaria RDT and 2.0% were coinfected by both microorganisms. Similar results were reported in Kinshasa in 2018, in a hospital-based survey, with an acute dengue frequency of 8.1% using dengue RDT and ELISA. Among 19 cases of acute dengue, 36.1% were positive to malaria; however, the frequency appeared slightly higher than the 24.2% reported in the current study among presented acute dengue fever [21].

In our study, in two households from different locations (the Kinshasa and Limete (Kingabwa) municipalities), the IgM and NS1 antigen were detected in two members of the same household, and both members presented similar clinical manifestations. This observation confirmed that human is the reservoir of this virus in an urban area, and the infection is actively transmitted between humans through infected mosquito bites in places where the competent vector exists [1]. In the Kinshasa municipality as well as in its neighboring municipalities (Lingwala, Barumbu) from Lukunga, *Ae. aegypti* was the predominant species. In 2022, cases of yellow fever outbreak were recorded in Kinshasa, Kimbanseke, precisely in the Kingasani health zones and Mont Ngafula municipalities [30]. The high demographical density with increasing population movement is known, among other factors, to spread infection to other areas. This observation is supported by the observation made during the wide yellow fever epidemic that occurred in Angola and the DRC in 2016 [47]. Similar to the geographical distribution of *Aedes*, the magnitude of human exposure varied also according to district and within the district from one municipality to another.

Considering the Tshangu district, of the blood samples tested in the Ndjili municipality, only one case was recorded using an NS1 RDT. However, this municipality was classified among those at high entomological risk of MBVD regarding the number of *Aedes*-positive container index and grid cells (44%). It is important to also the genetic population diversity and their competence to transmit different MBVDs. The competence varies according to the genetic population, which can also vary according to geographical location [48]. The Mont Amba district had a high number of seropositive cases. However, both *Aedes* species were recorded approximately at the same rate: *Ae. albopictus* slightly predominated in Kisenso. Matete (Maziba namely also De Marrais) and Limete (Kingabwa, Ndanu, Salongo, and Mombele areas), which are humid areas laid on the Ndjili and Congo Rivers, favorable conditions for mosquito multiplication. Early wide chikungunya outbreaks in 1999–2000 have occurred in Kingabwa, De Marrais, and Kikole in Nsele [49]. In these areas, *Aedes* should maintain their activity even during the dry season; this argument might explain the occurrence of active cases (NS1-, IgM-positive) recorded in the dry season. In the Funa district, in general, *Ae. aegypti* was predominant. Only IgG antibodies were detected among participants from the municipalities of Selembao and Bumbu, while cases of acute dengue were noted in the Kokolo military camp. However, the entomological baseline data was missed in the Bumbu municipality; during the inspection of the home place of the positive cases, we noted that automobile garage activities were widely undertaken in this municipality, leading to a high presence of discarded tires that should be the important source of mosquitoes. In Lukunga, the *Aedes* species’ composition varied according to the municipality. The *Ae. eagypti* and *Ae. albopictus* were recorded at approximately the same rate in Ngaliema, and *Ae. albopictus* slightly predominated where vegetation coverage appeared relatively high in Mont Ngafula and Ngaliema. The seropositivity was recorded as follows: The two positive cases from Gombe were recorded in a military camp built closer to the Congo River; this highly populated area is surrounded by many boat beaches, which connect Kinshasa with other provinces known at high risk of MBVD such as yellow fever [19]. The young age, the male sex, and the residence location adjusted to the participant’s marital status, occupation, and education level appeared as risk factors for dengue exposure occurrence.

These findings describe the population stratus that might be the priority for targeting control activities. Often, males are involved in professions that can expose them to increased mosquito bites such as those working in forested areas, automobiles mechanics, and military camps. Overall, four cases of acute dengue were recorded in both military camps involved in the current study. The militaries are among the social categories with an increasing trend of migration; in 1998, an outbreak of arboviruses occurred in a military camp in the province of Tshopo located in the northeastern part of the DRC [50]. The evidence of a higher prevalence of arboviruses among males has been documented in the literature [51]. The findings of this study also call for social mobilization, education programs to encourage the population to actively and persistently participate in the cleanliness of their living environment as it is within their reach, and adoption of protective behaviors against mosquito bites.

Despite the relevant findings reported in the current study, this study aimed solely aimed to gain insight into human exposure to dengue. This study had some limitations. The *Flavivirus* is known to present the cross-reactivity of IgG antibodies among these viruses’ family members [52], and the test specificity can be affected. The tests also detected IgM, and it has been coupled with the NS1 RDT, known as a more specific and sensitive marker for the primary cases of dengue. Therefore, the performance of our diagnostic maintained good consistency with available data from the literature [21,42,43,46]. Mostly due to a lack of study sufficient funds and time constraints, the performance of our diagnostic method was not compared to the standard diagnostic methods for the serology, such as enzyme-linked immunosorbent assays (ELISA) and the molecular detection of NS1 by RT-PCR. In addition, the number of participants tested was unequal among municipalities, with fewer participants in some municipalities. To facilitate the inclusion of the study and the blood sampling, the hospital-based survey and study design were used and allowed to include only febrile patients who attended the healthcare facilities. A community-based survey with a large sample size could provide more information related to the extent of human exposure.

## 5. Conclusions

The current study provides insights into epidemiological data on human risk exposure to dengue and its vectors and the burden of dengue among febrile patients above 5 years old. Dengue occurrence is linked to a certain degree to the geographical and ecological pattern of *Ae. aegypti* and *Ae. albopictus*. The distributions of both *Aedes* species appear to be influenced by environmental factors. These findings can assist the targeted control activities. These data are necessary for building rational approaches to better manage this common public health concern in sub-Saharan Africa as well as in the DRC.

## Figures and Tables

**Table 1:** Frequency of the positive grid cells stratified according to location and season in Kinshasa, DRC.

Variable		No Sampled Grid Cells *n*	Positive Grid Cells *n* (%)	(+) Cells in Rainy Season *n* (%)	(+) Cells in Dry Season *n* (%)
District	Municipality				
Funa	Selembao	100	17 (17.0)	12	5 (5.0)
	Ngiri-Ngiri	100	10 (10.0)	10 (10.0)	0 (0.0)
	Kasa-Vubu	100	9 (9.0)	9 (9.0)	0 (0.0)
	Bandalungwa	100	10 (10.0)	8 (8.0)	2 (2.0)
	Kalamu	100	13 (13.0)	13 (13.0)	0 (0.0)
Mont Amba	Ngaba	100	8 (8.0)	7 (7.0)	1 (1.0)
	Limete	100	32 (32.0)	32 (32.0)	2 (2.0)
	Matete	100	18 (18.0)	18 (18.0)	0 (0.0)
	Kisenso	100	21 (21.0)	21 (21.0)	2 (2.0)
Tshangu	Ndjili	100	44 (44.0)	44 (44.0)	3 (3.0)
	Kimbaseke	100	21 (21.0)	20 (20.0)	1 (1.0)
	Masina	100	24 (24.0)	24 (24.0)	0 (0.0)
	Nsele	100	12 (12.0)	12 (12.0)	1 (1.0)
	Maluku	50	13 (26.0)	13 (26.0)	1 (2.0)
Lukunga	Kinshasa	100	15 (15.0)	15 (15.0)	0 (0.0)
	Barumbu	100	20 (20.0)	20 (20.0)	0 (0.0)
	Lingwala	100	14 (14.0)	14 (14.0)	0 (0.0)
	Ngaliema	100	28 (28.0)	25 (25.0)	5 (5.0)
	Mont Ngafula	100	32 (32.0)	32 (32.0)	4 (4.0)
Total		1850	361 (19.5)	349 (18.9)	28 (1.5)

+: Positive.

**Table 2:** Frequency of the different types of the positive grid cells stratified according to location in Kinshasa, DRC.

Municipality	N	Bamboo *n* (%)	Discarded Container *n* (%)	Container for Domestic Uses *n* (%)	Tire *n* (%)
Ngaliema	53	2 (3.8)	12 (22.6)	30 (56.6)	9 (17.0)
Kinshasa	23	0 (0.0)	15 (65.2)	4 (17.4)	4 (17.4)
Barumbu	39	0 (0.0)	27 (69.2)	4 (10.3)	8 (20.5)
Lingwala	21	0 (0.0)	11 (52.4)	6 (28.6)	4 (19.0)
Mont Ngafula	71	5 (7.0)	3 (4.2)	54 (76.1)	9 (12.7)
Maluku	24	1 (4.1)	3 (12.6)	15 (62.5)	5 (20.8)
Nsele	16	4 (25.0)	2 (12.5)	8 (50.0)	2 (12.5)
Kimbanseke	34	6 (17.6)	7 (20.7)	13 (38.2)	8 (23.5)
Masina	40	3 (7.5)	8 (20.0)	12 (30.0)	17 (42.5)
Ndjili	80	1 (1.3)	18 (22.5)	8 (10.0)	53 (66.2)
Kasa-Vubu	23	0 (0.0)	2 (8.7)	3 (13.0)	18 (78.3)
Ngiri-Ngiri	15	0 (0.0)	4 (26.7)	3 (20.0)	8 (53.3)
Bandal	19	0 (0.0)	2 (10.5)	4 (21.1)	13 (68.4)
Selembao	49	0 (0)	0 (0.0)	27 (55.1)	22 (44.9)
Kalamu	20	2 (10.0)	8 (40.0)	1 (5.0)	9 (45.0)
Kisenso	37	1 (2.7)	6 (16.2)	27 (73.0)	3 (8.1)
Ngaba	13	0 (0.0)	2 (15.4)	2 (15.4)	9 (69.2)
Limete	65	1 (1.5)	18 (27.7)	10 (15.4)	36 (55.4)
Matete	39	0 (0.0)	13 (33.3)	4 (10.3)	22 (56.4)
Total	681	26 (3.9)	161 (23.6)	235 (34.5)	259 (38.0)

**Table 3:** Frequency of the positive grid cells of *Aedes aegypti* and *Aedes albopictus* stratified according to location and season in Kinshasa, DRC.

Municipality	No Sampled Grid Cells	Rainy Season		Dry Season	
		*Ae. aegypti*	*Ae. albopictus*	*Ae. aegypti*	*Ae. albopictus*
Selembao	100	8 (8.0)	7 (7.0)	4 (4.0)	1 (1.0)
Ngiri-Ngiri	100	7 (7.0)	3 (3.0)	0 (0.0)	0 (0.0)
Kasa-Vubu	100	6 (6.0)	3 (3.0)	0 (0.0)	0 (0.0)
Bandalungwa	100	8 (8.0)	0 (0.0)	1 (1.0)	1 (1.0)
Kalamu	100	10 (10.0)	4 (4.0)	0 (0.0)	0 (0.0)
Ngaba	100	4 (4.0)	3 (3.0)	0 (0.0)	1 (1.0)
Limete	100	16 (16.0)	18 (18.0)	0 (0.0)	2 (2.0)
Matete	100	11 (11.0)	9 (9.0)	0 (0.0)	0 (0.0)
Kisenso	100	15 (15.0)	8 (8.0)	2 (2.0)	0 (0.0)
Ndjili	100	24 (24.0)	26 (26.0)	2 (2.0)	1 (1.0)
Kimbaseke	100	4 (4.0)	16 (16.0)	0 (0.0)	1 (1.0)
Masina	100	10 (10.0)	17 (17.0)	0 (0.0)	0 (0.0)
Nsele	100	2 (2.0)	10 (10.0)	0 (0.0)	1 (1.0)
Maluku	100	3 (6.0)	10 (20.0)	0 (0.0)	1 (2.0)
Kinshasa	100	14 (14.0)	4 (4.0)	0 (0.0)	0 (0.0)
Barumbu	100	20 (20.0)	0 (0.0)	0 (0.0)	0 (0.0)
Lingwala	100	13 (13.0)	2 (2.0)	0 (0.0)	0 (0.0)
Ngaliema	100	17 (17.0)	8 (8.0)	2 (2.0)	3 (3.0)
Mont Ngafula	100	16 (16.0)	21 (21.0)	2 (2.0)	2 (2.0)
Total	1850	208 (11.2)	169 (9.1)	13 (0.7)	14 (0.8)

**Table 4:** Sociodemographic characteristics of the participants.

Variable	No. of Respondents (%)
**Age Group**	
6–17 years	89 (22.2)
18–34 years	147 (36.8)
35–51 years	87 (21.7)
≥52 years	77 (19.3)
**Sex**	
Male	155 (38.8)
Female	245 (61.2)
**Marital status**	
Unmarried	235 (58.7)
Married	165 (41.3)
**Education level**	
None	38 (9.5)
Primary	117 (29.2)
Secondary	164 (41.0)
University	81 (20.3)
**Occupation**	
Military	18 (4.5)
Faming crop	18 (4.5)
Health professional	11 (2.8)
Trading	14 (3.5)
Student	108 (27.1)
Housewife	65 (16.3)
Other	26 (6.6)
None	139 (34.7)

**Table 5:** Frequencies of dengue seropositivity and malaria using the RDTs among febrile patients in Kinshasa, 2022.

Variable		Samples Teste (N = 400)	Seropositive Samples *n* (%)	Dengue RDT Abs *n* (%)		Malaria RDT *n* (%)
District	Municipality			Ig G+	Ig M+	*P. falciparum*
Tshangu		100	6 (6.0)	4 (4.0)	4 (4.0%)	30 (30.0)
	Kimbanseke	30	1 (3.3)	1 (3.3)	1 (3.3)	12 (40.0)
	Masina	21	1 (4.8)	1 (4.8)	0 (0.0)	4 (19.0)
	Ndjili	30	0 (0.0)	0 (0.0)	0 (0.0)	9 (30.0)
	Nsele	19	4 (21.4)	2 (10.5)	3 (15.8)	5 (26.3)
Mont Amba		100	22 (22.0)	10 (10.0)	17 (17.0)	30 (30.0)
	Lemba	4	0 (0.0)	0 (0.0)	0 (0.0)	1 (25.0)
	Matete	24	5 (20.8)	1 (4.2)	4 (16.6)	5 (20.8)
	Kisenso	28	6 (21.4)	3(10.7)	4(14.3)	11 (39.3)
	Ngaba	7	0 (0.0)	0 (0.0)	0 (0.0)	6 (85.7)
	Limete	37	11 (29.7)	6 (16.2)	9 (24.3)	7 (18.9)
Funa		100	17 (17.0)	15 (15.0)	2 (2.0)	23 (23.0)
	Kalamu	12	1 (8.3)	1 (8.3)	0 (0.0)	5 (41.7)
	Kasa-Vubu	10	3 (30.0)	3 (30.0)	0 (0.0)	1 (10.1)
	Ngiri-Ngiri	10	0 (0.0)	0 (0.0)	0 (0.0)	2 (20.0)
	Bandal	18	2 (11.1)	0 (0.0)	2 (11.1)	3 (16.7)
	Selembao	29	7 (24.1)	7 (24.1)	0 (0.0)	7 (24.1)
	Bumbu	21	4 (19.1)	4 (19.1)	0 (0.0)	5 (23.8)
Lukunga		100	14 (14.0)	9 (9.0)	8 (8.0)	33.(33.0)
	Kinshasa	14	2 (14.3)	1 (7.1)	1 (7.1)	3 (21.4)
	Barumbu	17	1 (5.9)	1 (5.9)	0 (0.0)	6 (5.9)
	Gombe	16	2 (12.5)	0 (0.0)	2 (12.5)	6 (37.5)
	Ngaliema	30	3. (10.0)	3. (10.0)	2 (6.8)	14 (46.6)
	Mont Ngafula	23	6 (26.1)	4 (17.4)	3 (13.0)	9 (39.1)
Total		400	59 (14.8)	39 (9.8)	31 (7.8)	116 (29.2)

**Table 6:** Frequency of dengue NS1-positive cases stratified according to the serological status of the study participants.

Variable Serological Status	Samples Tested (*n* = 100)	NS1 Positive *n* (%)
IgM	21	18 (85.7)
IgG	28	4 (16.0)
IgG and Ig M	10	0 (0.0)
Negative	41	2 (4.8)

**Table 7:** Clinical manifestations of acute fever of the study participants stratified by DENV and malaria infection in Kinshasa.

Variable Clinical Symptoms	Total (*n* = 400)	Acute Dengue (*n* = 37)
Headaches	204 (51.4)	23 (62.2)
Joints pain	112 (28.2)	13 (35.1.)
Muscles pain	74 (18.6)	10 (27.0)
Back pain	12 (3.0)	-
Abdominal pain	13 (3.3)	1 (2.7)
Rash	15 (3.8)	-
Vomiting	45 (11.3)	6 (16.2)
Diarrhea	22 (5.5)	3 (8.1)
Cough	12 (3.0)	-
Fatigue	8 (2.0)	-
Jaundice	5 (1.3)	-
Other	11 (2.7)	-

**Table 8:** Sociodemographic risks factors associated to human dengue exposure in the current study, Kinshasa 2022.

Variable	OR	95%CI	*p* < 0.05	ORa	95%CI	*p* < 0.05
**Age Group**						
6–17 years	1			1		
18–34 years	0.9	0.4–1.9	0.954	0.8	0.3–1.9	0.0252
35–51 years	0.5	0.2–1.6	0.151	0.3	0.08–0.8	0.697
52 years	0.6	0.2–1.2	0.379	0.3	0.09–0.9	0.0489
**Sex**						
Female	1		0	1		
Male	1.3	0.7–2.2	0.3380	2.4	1.1–5.2	0.0271
**Marital status**						
Unmarried	1			1		
Married	0.8	0.4–1.4	0.541	1.3	0.6–2.6	0.412
**Education level**						
None	1			1		
Primary	0.6	0.2–1.5	0.302	0.6	0.2–1.7	0.378
Secondary	0.4	0.2–1.1	0.118	0.5	0.2–1.4	0.2414
University	0.5	0.1–1.3	0.174	0.4	0.1–1.3	0.1462
**Occupation**						
None	1			1		
Yes	0.7	0.4–1.2	0.208	0.7	0.3–1.5	0.420
**District**						
Tshangu	1			1		
Mont amba	3.7	1.5–9.2	0.004	3.2	1.2–8.2	0.0123
Funa	2.7	1.07–6.8	0.034	2.1	0.7–5.6	0.1375
Lukunga	2.3	0.9–6.0	0.076	1.9	0.7–5.4	0.1852

## Data Availability

All data supporting the study findings are included in this published article.

## Abbreviations

MBD: Mosquito borne disease, MBVD: Mosquito borne viral disease, ELISA: enzyme-linked immunosorbent assays, RT–PCR: reverse transcriptase–polymerase chain reaction, RDT: rapid diagnostic test, DRC: Democratic Republic of the Congo.

## References

[1] WHO (2022). Dengue and Severe Dengue.

[2] WHO (2021). World-Malaria-Report-2021.

[3] Nana-Ndjangwo S.M., Djiappi-Tchamen B., Mony R., Demanou M., Keumezeu-Tsafack J., Bamou R., Awono-Ambene P., Bilong Bilong C.F., Antonio-Nkondjio C. (2022). Assessment of Dengue and Chikungunya Infections among Febrile Patients Visiting Four Healthcare Centres in Yaoundé and Dizangué, Cameroon. Viruses.

[4] Rugarabamu S., Rumisha S.F., Mwanyika G.O., Sindato C., Lim H.-Y., Misinzo G., Mboera L.E.G. (2022). Viral haemorrhagic fevers and malaria co-infections among febrile patients seeking health care in Tanzania. Infect. Dis. Poverty.

[5] Siribhadra A., Ngamprasertchai T., Rattanaumpawan P., Lawpoolsri S., Luvira V., Pitisuttithum P. (2022). Antimicrobial Stewardship in Tropical Infectious Diseases: Focusing on Dengue and Malaria. Trop. Med. Infect. Dis..

[6] Huang Y.-J.S., Higgs S., Vanlandingham D.L. (2017). Biological Control Strategies for Mosquito Vectors of Arboviruses. Insects.

[7] Girard M., Nelson C.B., Picot V., Gubler D.J. (2020). Arboviruses: A global public health threat. Vaccine.

[8] Oragwa A.O., Oragwa F.C., Oluwayelu D.O. (2022). Serologic evidence of silent Rift Valley fever virus infection among occupationally exposed persons in northern Nigeria. J. Infect. Dev. Ctries..

[9] Kanunfre K.A., Rocha M.C., Malta M.B., de-Souza R.M., Castro M.C., Boscardin S.B., Souza H.F.S., Witkin S.S., Cardoso M.A., Okay T.S. (2022). Silent circulation of Chikungunya virus among pregnant women and newborns in the Western Brazilian Amazon before the first outbreak of chikungunya fever. Rev. Inst. Med. Trop. São Paulo.

[10] Jing Q., Wang M. (2019). Dengue epidemiology. Glob. Health J..

[11] Bettis A.A., Jackson M.L., Yoon I.-K., Breugelmans J.G., Goios A., Gubler D.J., Powers A.M. (2022). The global epidemiology of chikungunya from 1999 to 2020: A systematic literature review to inform the development and introduction of vaccines. PLoS Negl. Trop. Dis..

[12] Mbanzulu K.M., Mboera L.E.G., Luzolo F.K., Wumba R., Misinzo G., Kimera S.I. (2020). Mosquito-borne viral diseases in the Democratic Republic of the Congo: A review. Parasites Vectors.

[13] Ido E., Ahuka S., Karhemere S., Shibata K., Kameoka M., Muyembe J.J. Dengue Virus Infection during an Outbreak of Chikungunya Virus in Democratic Republic of Congo, Annales Africaines de Médecine. https://anafrimed.net.

[14] Proesmans S., Katshongo F., Milambu J., Fungula B., Muhindo H.M., Ahuka-Mundeke S., Luz R.I.D., Esbroeck M.V., Arien K.K., Cnops L. (2019). Dengue and chikungunya among outpatients with acute undifferentiated fever in Kinshasa, Democratic Republic of Congo: A cross sectional study. PLoS Negl. Trop. Dis..

[15] Willcox A.C., Mumba D., Jadi R., de Silva A.M., Collins M.H., Meshnick S.R., Tshefu A., Parr J.B., Keeler C., Kashamuka M. (2018). Seroepidemiology of dengue, Zika, and yellow fever viruses among children in the Democratic Republic of the Congo. Am. J. Trop. Med. Hyg..

[16] Makiala-Mandanda S., Ahuka-Mundeke S., Abbate J.L., Pukuta-Simbu E., Nsio-Mbeta J., Berthet N., Leroy E.M., Becquart P., Muyembe-Tamfum J.-J. (2018). Identifcation of dengue and chikungunya cases among suspected cases of yellow fever in the Democratic Republic of the Congo. Vector Borne Zoonotic Dis..

[17] Näslund J., Ahlm C., Islam K., Evander M., Bucht G., Lwande O.W. (2021). Emerging Mosquito-Borne Viruses Linked to Aedes aegypti and Aedes albopictus: Global Status and Preventive Strategies. Vector Borne Zoonotic Dis..

[18] Matysiak A., Roess A. (2017). Interrelationship between climatic, ecologic, social, and cultural determinants affecting dengue emergence and transmission in Puerto Rico and their implications for Zika response. J. Trop. Med..

[19] Robert M.A., Stewart-Ibarra A.M., Estallo E.L. (2020). Climate change and viral emergence: Evidence from *Aedes*-borne arboviruses. Curr. Opin. Virol..

[20] Kolimenakis A., Heinz S., Wilson M.L., Winkler V., Yakob L., Michaelakis A., Papachristos D., Richardson C., Horstick O. (2021). The role of urbanisation in the spread of *Aedes* mosquitoes and the diseases they transmit—A systematic review. PLoS Negl. Trop. Dis..

[21] Dalpadado R., Amarasinghe D., Gunathilaka N., Ariyarathna N. (2022). Bionomic aspects of dengue vectors *Aedes aegypti* and *Aedes albopictus* at domestic settings in urban, suburban and rural areas in Gampaha District, Western Province of Sri Lanka. Parasites Vectors.

[22] Bobanga T., Moyo M., Vulu F., Irish S.R. (2018). First Report of *Aedes albopictus* (Diptera: Culicidae) in the Democratic Republic of Congo. Afr. Entomol..

[23] Wat’senga F.T., Fasine S., Manzambi E.Z., Marquetti M.C., Binene G.M., Ilombe G., Mundeke R.T., Smitz N., Bisset J.A., Van W.B. (2021). High *Aedes* spp. larval indices in Kinshasa, Democratic Republic of Congo. Parasites Vectors.

[24] Mbanzulu K.M., Mboera L.E.G., Wumba R., Engbu D., Bojabwa M.M., Zanga J., Mitashi P.M., Misinzo G., Kimera S.I. (2022). Physicochemical characteristics of *Aedes* mosquito breeding habitats in suburban and urban areas of Kinshasa, Democratic Republic of the Congo. Front. Trop. Dis..

[25] Mariën J., Laurent N., Smitz N., Gombeer S. (2022). First observation of *Aedes albopictus* (Skuse, 1894) (Diptera: Culicidae) in Tshuapa province (Boende), Democratic Republic of the Congo African. Entomology.

[26] De Weggheleire A., Nkuba-Ndaye A., Mbala-Kingebeni P., Mariën J., Kindombe-Luzolo E., Ilombe G., Mangala-Sonzi D., Binene-Mbuka G., De Smet B., Vogt F. (2021). A Multidisciplinary Investigation of the First Chikungunya Virus Outbreak in Matadi in the Democratic Republic of the Congo. Viruses.

[27] Otshudiema J.O., Ndakala N.G., Mawanda E.K., Tshapenda G.P., Kimfuta J.M., Nsibu L.N., Gueye A.S., Dee J., Philen R.M., Giese C. (2017). Yellow fever outbreak Kongo Central province. Democratic Republic of the Congo. Morb. Mortal. Wkly. Rep..

[28] WHO Yellow-Fever in West and Central-Africa.

[29] Hasan M.J., Tabassum T., Sharif M., Khan M., Saeed A., Bipasha A.R., Basher A., Islam M.R., Amin M.R. (2021). Comparison of clinical manifestation of dengue fever in Bangladesh: An observation over a decade. BMC Infect. Dis..

[30] Liu L.-T., Chen C.-H., Tsai C.-Y., Lin P.-C., Hsu M.-C., Huang B.-Y., Wang Y.-H., Tsai J.-J. (2020). Evaluation of rapid diagnostic tests to detect dengue virus infections in Taiwan. PLoS ONE.

[31] Lim J.K., Alexander N., Tanna G.L.D. (2017). A systematic review of the economic impact of rapid diagnostic tests for dengue. BMC Health Serv. Res..

[32] Wikipedia Kinshasa. The Free Encyclopedia. https://en.wikipedia.org/wiki/Kinshasa.

[33] Focks D.A., UNDP/World Bank/WHO Special Programme for Research and Training in Tropical Diseases (2004). A Review of Entomological Sampling Methods and Indicators for Dengue Vectors.

[34] Rueda L.M. (2004). Pictorial Keys for the Identification of Mosquitoes (Diptera: Culicidae) Associated with Dengue Virus Transmission. Zootaxa.

[35] Santoso M.S., Yohan B., Denis D., Hayati R.F., Haryanto S., Trianty L., Noviyanti R., Hibberd M.L., Sasmono R.T. (2020). Diagnostic accuracy of 5 different brands of dengue virus non-structural protein 1 (NS1) antigen rapid diagnostic tests (RDT) in Indonesia. Diagn. Microbiol. Infect. Dis..

[36] Alidjinou E.K., Tardieu S., Vrenken I., Hober D., Gourinat A.-C. (2022). Prospective Evaluation of a Commercial Dengue NS1 Antigen Rapid Diagnostic Test in New Caledonia. Microorganisms.

[37] Dhar-Chowdhury P., Haque C.E., Lindsay R., Hossain S. (2016). Socioeconomic and Ecological Factors Influencing *Aedes aegypti* Prevalence, Abundance, and Distribution in Dhaka, Bangladesh. Am. J. Trop. Med. Hyg..

[38] Ngugi H.N., Mutuku F.M., Ndenga B.A.M., Usunzaji P.S., Mbakaya J.O., Aswani P., Irungu L.W., Mukoko D., Vulule J., Kitron U. (2017). Characterization and productivity profiles of *Aedes aegypti* (L.) breeding habitats across rural and urban landscapes in western and coastal Kenya. Parasites Vectors.

[39] Rasheed S.B., Boots M., Frantz A.C., Butlin R.K. (2013). Population structure of the mosquito *Aedes aegypti* (*Stegomyia aegypti*) in Pakistan. Med. Vet. Entomol..

[40] Mohammadi A., Mostafavi E., Zaim M., Enayati A., Basseri H.R., Mirolyaei A.R., Poormozafari J., Gouya M.M. (2022). Imported tires; a potential source for the entry of *Aedes* invasive mosquitoes to Iran. Travel Med. Infect. Dis..

[41] Soares A.P.M., Ingrid N.G., Rosário I.N.G., Silva I.M. (2020). Distribution and preference for oviposition sites of *Aedes albopictus* (Skuse) in the metropolitan area of Belém, in the Brazilian Amazon. J. Vector Ecol..

[42] Camargo C., Alfonso-Parra C., Díaz S., Rincon D.F., Ramírez-Sánchez L.F., Agudelo J., Barrientos L.M., Villa-Arias S., Avila F.W. (2021). Spatial and temporal population dynamics of male and female *Aedes albopictus* at a local scale in Medellín, Colombia. Parasites Vectors.

[43] Gainor E.M., Harris E., LaBeaud A.D. (2022). Uncovering the Burden of Dengue in Africa: Considerations on Magnitude, Misdiagnosis, and Ancestry. Viruses.

[44] Pal S., Dauner A.L., Mitra I., Forshey B.M., Garcia P., Morrison A.C., Halsey E.S., Kochel T.J., Wu S. (2014). Evaluation of Dengue NS1 Antigen Rapid Tests and ELISA Kits Using Clinical Samples. PLoS ONE.

[45] Liu L.-T., Dalipanda T., Jagilly R., Wang Y.-H., Lin P.-C., Tsai C.-Y., Lin P.-C., Tsai C.-Y., Lai W.-T., Tsai J.-J. (2018). Comparison of two rapid diagnostic tests during a large dengue virus serotype 3 outbreak in the Solomon Islands in 2013. PLoS ONE.

[46] Blacksell S.D., Jarman R.G., Bailey M.S., Tanganuchitcharnchai A., Jenjaroen K., Gibbons R.V., Paris D.H., Premaratna R., Janaka de Silva H., Lalloo D.G. (2011). Evaluation of six commercial point-of-care tests for diagnosis of acute dengue infections: The need for combining NS1 antigen and IgM/IgG antibody detection to achieve acceptable levels of Accuracy. Clin. Vaccine Immunol..

[47] Kraemer M.U., Faria N.R., Reiner R.C., Golding N., Nikolay B., Stasse S., Johansson M.A., Salje H., Faye O., Wint G.W. (2017). Spread of yellow fever virus outbreak in Angola and the Democratic Republic of the Congo 2015–16: A modelling study. Lancet Infect. Dis..

[48] Paupy C., Chantha N., Reynes J.M., Failloux A.B. (2005). Factors influencing the population structure of *Aedes aegypti* from the main cities in Cambodia. Heredity.

[49] Muyembe-Tamfum J.J., Peyrefitte C.N., Yogolelo R., Basisya E.M., Koyange D., Pukuta E., Mashako M., Tolou H., Durand J.P. (2003). Epidemic of Chikungunya virus in 1999 and 200 in the Democratic Republic of the Congo. Med. Trop. Rev. Corps. Sante. Colon..

[50] Nur Y.A., Groen J., Heuvelmans H., Tuynman W., Copra C., Osterhaus A.D. (1999). An outbreak of West Nile fever among migrants in Kisangani, Democratic Republic of Congo. Am. J. Trop. Med. Hyg..

[51] Power G.M., Vaughan A.M., Clemente N.S., Pescarini J.M., Paixão E.S., Lobkowicz L., Raja A.I., Souza A.P., Barreto M.L., Brickley E.B. (2022). Socioeconomic risk markers of arthropod-borne virus (arbovirus) infections: A systematic literature review and meta-analysis. BMJ Glob. Health.

[52] Hou B., Chen H., Gao N., An J. (2022). Cross-Reactive Immunity among Five Medically Important Mosquito-Borne Flaviviruses Related to Human Diseases. Viruses.

